# The impact of the mountain barrier on the spread of heavy metal pollution on the example of Gorce Mountains, Southern Poland

**DOI:** 10.1007/s10661-022-10316-0

**Published:** 2022-08-10

**Authors:** Paweł Miśkowiec

**Affiliations:** Department of Environmental Chemistry, Faculty of Chemistry, Jagiellonian University, Gronostajowa 2, 30-387 Krakow, Poland

**Keywords:** Zinc, Lead, Cadmium, Mountain area, Spread of pollution

## Abstract

The main objective of this study was to determine the content, mobility, and the variability of concentration of zinc, lead, and cadmium in soils from the Gorce Mountains (south Poland), located over 100 km south-east from the potential industrial sources of contamination—zinc-lead sulfide ore mine and smelter in Bukowno, as well as hard coal mines of Silesia region and Kraków Nowa-Huta steelwork. The abovementioned problem is crucial in the context of the traditional mountain farming still extant in the region, as well as intensively developing tourism. The geoaccumulation index and potential ecological risk index were adopted to evaluate soil pollution in the study area and the BCR sequential extraction technique to assess mobility of the abovementioned elements. The obtained results clearly show that the pollution from distant industrial sources in the mountains is detectable. Apart from the increased concentrations of the tested metals in the soil (especially available forms), there is also a strong correlation between the concentrations of lead, zinc, and cadmium, which proves their common source of origin. The main evidence is the fact that differences in the concentrations of the tested metals on the windward and leeward sides were statistically significant. This also means that the studied mountain area, despite relatively low altitudes (up to 1310 m above sea level), constitutes a measurable barrier to the spread of atmospheric pollutants.

**Supplementary Information:**

The online version contains supplementary material available at 10.1007/s10661-022-10316-0.

## Introduction

The human pressure on the mountain ranges is the serious problem in many regions all over the world (Elsen et al., 2020). This is due to the fact that mountains are by definition extremely fragile ecosystems because of poorly developed soils and usually harsh weather conditions. As a result, mountain biocenoses are particularly susceptible to environmental changes (FAO-UNCCD, 2019; Tsering et al., 2010). One of the main environmental impacts of industry is the progressive change in the chemical composition of such ecosystems in delicate balance, located even far from emission sources. One example of such negative impact is the emission of heavy metals (Kabata-Pendias, 2010). Continuous release of these toxicants from anthropogenic sources causes significant changes in the biogeochemical cycle of the elements (Dang et al., 2021; Kabata-Pendias, 2010; Opekunova et al., 2020). Even though they are often considered as a local issue, heavy metals when adsorbed on air-borne particles or dissolved in atmospheric water droplets can be transported in the atmosphere for long distances (Poličnik et al., 2004). The studied metals, cadmium, lead, and zinc, are emitted to the environment mainly by industrial combustion processes. Another potentially significant source of the abovementioned metals in the environment is the massive use of chemical fertilizers as well as some types of pesticides (Das et al., 1997; Liao et al., 2019; Miśkowiec, 2018a; Wang et al., 2021; Xing et al., 2020; Yang et al., 2020). Mountain ranges extending across the direction of pollution movement create the additional natural blockage of tropospheric air movement. This leads to higher precipitation in the area. Thus, mountain massifs may be a barrier for spreading of pollutants and simultaneously they can be more exposed to pollution loads than adjacent lower located regions (Bytnerowicz et al., 2003; Zsigmond & Urák, 2011). Mountain slopes, depending on their exhibition, may differ in both dominating wind direction and intensity, and hence possibly they can be exposed to pollution in different intensity (Klimek et al., 2015).

In the case of the studied area, the main long-range emission source of the pollution is the Jaworznicko-Chrzanowski Industrial Belt, situated in the west and north west of the Małopolska Province, the south Poland, and the Krakow Industrial District located in the central part of the voivodeship. Ore mining and smelting in the region started in the twelfth century; however, they intensified significantly with the outbreak of the industrial revolution (Cabala & Teper, 2007). The largest emission source of non-ferrous heavy metals is the Bolesław Mining and Metallurgy Company, which pollutes the environment both with the by-products of previous mining activities, stored in ore heaps, and with air pollution from combustion processes and other technological processes during the production of various forms of zinc from metalliferous (mainly zinc-lead sulfide) ores (Chrastný et al., 2012; Gruszecka-Kosowska & Kicińska, 2017; Miśkowiec, 2018b; Miśkowiec et al., 2015). The specific metal assemblage of the pollutants, with domination of Pb, Zn, and Cd, is inherited from primary carbonate-hosted lead–zinc ore deposits related to Mississippi Valley-Type (MVT) formation, that is mined and smelted (Cabala & Teper, 2007). The influx of toxic substances from industrial plants (hard coal mining) situated several kilometers further to the west in the Silesian conurbation may be also significant (Dudka et al., 1995; Loska et al., 2004). According to our previous studies, the impact of the abovementioned emitters is visible at least 30–40 km around (Miśkowiec, 2018b; Miśkowiec et al., 2014). However, there were some premises on much more distant influence, especially on the southern located mountain regions (Józefowska et al., 2014; Małek et al., 2018).

Another potential source of pollution that is difficult to ignore is the metallurgical plant in Kraków Nowa Huta and cooperating companies (like coking plant and foundry companies). This plant and associated companies have contributed to the overall emission of gasses and dust since the 1950s. This has been confirmed by a series of studies of soils conducted in and around Krakow (Bokwa, 2008; Mundała et al., 2013). Nowadays, the processes that can have the greatest impact on the emission from the Krakow plants are coking, hot rolling, and foundry (mainly electric arc furnace casting) (Da Silva et al., 2008). However, discussing Nowa Huta steelwork, it should be emphasized that its location almost exactly to the north and north-east of the studied area means that, with the prevailing western winds, the impact of the plant in this case may be slightly smaller than it could only result from comparing of the size of the emitters (IMGW, 2021). Moreover, it should be highlighted that the restrictive emission standards for industry in force for over 30 years in Poland together with a number of actions taken by the inhabitants and the local government of Krakow, as well as the reduction of production (e.g., closing the blast furnace), contributed to a significant decrease in emissions from these sources (Czarnecka et al., 2021).

Finally, the image of land pollution is also influenced by local emission sources—the so-called low emission from the towns surrounding Gorce, as well as popular access roads. However, the nature of these pollutants is slightly different from the discussed industrial pollutants, which does not change the fact that they may complicate the overall picture of the state of environment.

Gorce Mountains are placed around 80 km from Kraków and over 100 km from the described earlier industrial belt—potential sources of industrial contamination. Despite the distance, it is practically the first significant elevation of the terrain in the south-east direction from pollution emitters. Moreover, the Gorce Mountains have been already the object of interest of researchers dealing with the state of the environment since the end of 1980s, due to the noted air pollution with dust, sulfur, and nitrogen oxides which was probably one of the causes of changes in biocenoses, including the dying out of mountain spruce forests (Godzik, 1991; Niemtur, 1997; Tanona & Czarnota, 2020).

In case of this region, the problem of contamination is crucial in context of both the threat to natural mountain ecosystems and traditional mountain farming still extant in the region, as well as intensively developing tourism (Bucała, 2014).

Thus, the aim of the study was to compare the concentration and mobility of metals in meadow and glade soils situated on windward and leeward side of the mountain massif and to evaluate the relationship between the distance from the emission sources and retention as well as availability of the metals in soil. The study assessed the role of the mountain barrier with low relative height and thus with non-obvious features of the obstacle, in the long-range spread of pollution. Moreover, the potential risk related to the accumulation of heavy metals in the examined mountain soils was assessed as well.

## Materials and methods

### Characterization of the Gorce Mountains

**Fig. 1:**
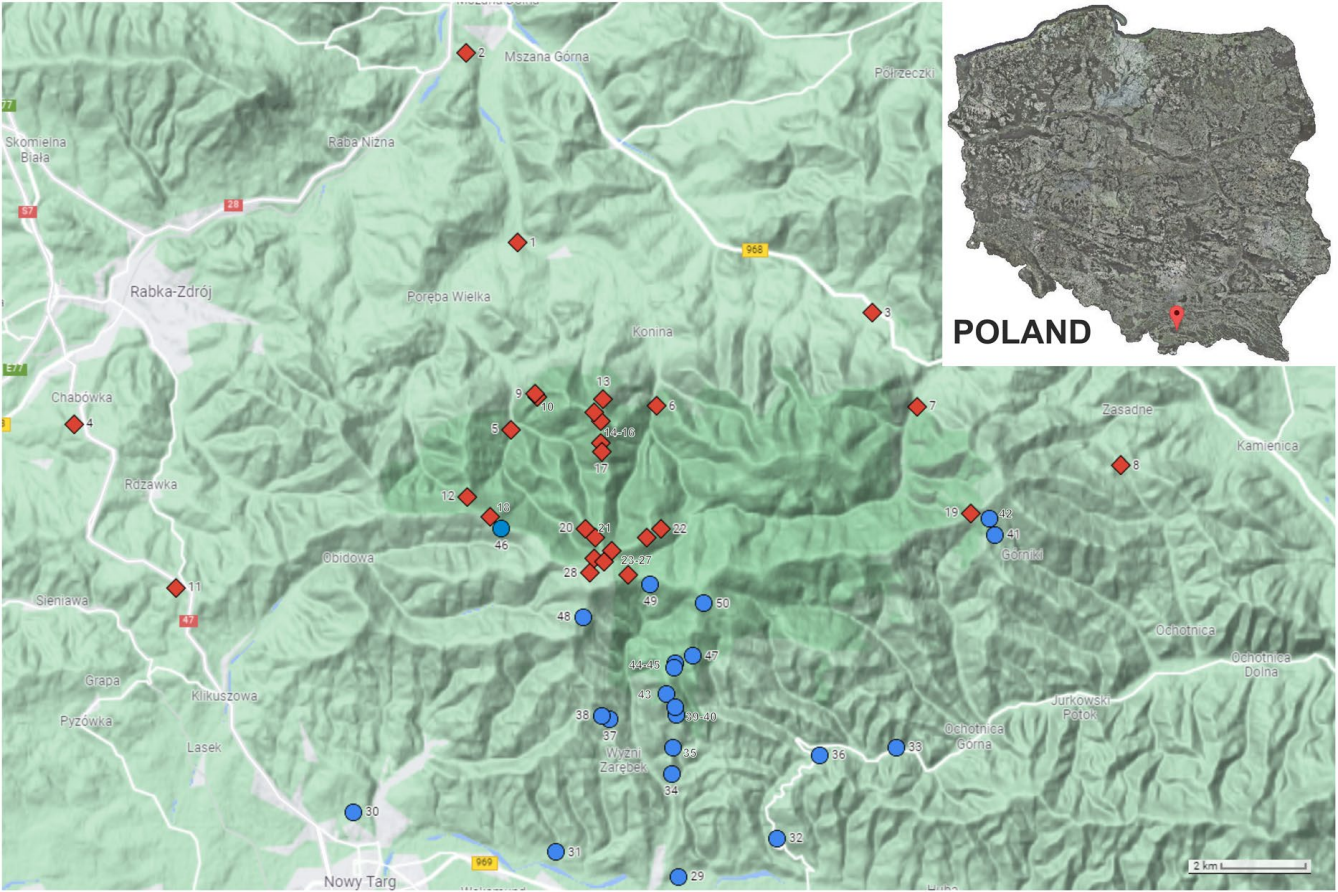
Localization of the sampling points in Gorce Mountains, south Poland. Sampling points from the windward side are marked as red squares, and samples points on the leeward side are marked as blue circles. A more detailed part of the map on a larger scale of the area with compacted measuring points is added in the supporting material as Fig. S1. Copyright Google 2022

Gorce Mountains are part of the Carpathian Mountains—the second biggest mountain range in Europe after the Alps. This mesoregion covers an area of about 578 km^2^, located within the western Carpathians. The highest peak of the mountain group is Turbacz with a height of 1310 m above sea level (m a.s.l.). Seven distinct mountain ridges diverge from Turbacz in different directions, as a result of which this mountain group forms a characteristic starry expanse cut by deep river valleys (Fig. 1). Gorce Mountains are characterized by considerable relative heights. Thanks to the relief described above, the massif constitutes a clear and compact barrier to the west and north-west winds dominating in the region (IMGW, 2021). The building blocks of Gorce are the Carpathian flysch formed by rootles (detached from their substratum) nappes, consisting of series of alternating marine deposits of sandstones, shales, and claystones built of clay minerals, quartz, feldspar, muscovite, and similar minerals (Golonka et al., 2021; Kroh & Pawlik, 2021). Therefore, it is not a significant source of the tested heavy metals (Bońda & Brzeziński, 2017; Cieszkowski et al., 2017; Szczęch & Cieszkowski, 2021). In sandstone outcrops, there are mainly acidic or leached brown soils (Miechówka et al., 2006). They are mainly light or medium clays, in most cases dusty. In the outcrops of slate, there are lessive soils, which in their mechanical composition are characterized by a high content of dusty fraction. In the upper parts of the Gorce Mountains, on a poor, sandy ground, podzolic soils developed. The soils are mostly poorly developed and shallow, with a high proportion of skeletal fraction in the entire soil profile (Bucała, 2014; Bucała et al., 2016). Forests cover about 65% of the mountains. Characteristic for this massif is the numerous glades and meadows resulting from centuries of grazing of sheep and cattle. Within this mountain group, the Gorce National Park was created in 1981. The park covers an area of 70.30 km^2^, which is 12.2% of the Gorce area (Kroh & Pawlik, 2021).

### Field sampling

In the years 2017–2020, the measurements of zinc, lead, and cadmium in soils of the open areas (meadows, glades, pastures, and agriculture lands) and in the selected areas of the Gorce Mountains were conducted. The samples were collected from 50 points in a whole Gorce area. All the samples were collected from arable and grasslands situated either in the National Park or in the Park’s buffer zone. The samples were collected up to the depth of 0.3 m below land surface (topsoil). The sampling points were chosen on the north and north-west – windward slopes (marked as red squares in the Fig. 1) and on the south and south-east – leeward slopes (marked with blue circles in the Fig. 1). In five points (#1, 4, 8, 31, 36), additional samples from substratum/bedrock horizon were collected, to assess the possible influence of the parent rock on the results obtained. The sampling points are described in detail in the supplementary material (Table S1 and Table S2).

In every sampling area, 10 subsamples were collected from the square of approximately size 10 × 10 m and mixed up to give a bulk sample, weighting up to 1 kg. The bulk soil samples were air-dried, crushed, and sieved through a sieve with a mesh size of 2 mm. Afterwards, every primary sample was divided in four equal parts. Three-quarters of each sample was discarded and the remained quarter called laboratory sample was used for the analysis.

### Determination of the soil physicochemical properties and heavy metal analysis

The basic soil parameters were studied such as pH, the amount of clay fraction, and organic matter, to assess the potential susceptibility to contamination with heavy metals.

The soil pH was measured in 1.0-M potassium chloride solution, pursuant to ISO norm no. 10390:2005 (ISO, 2005), using an Elmetron CP-401 pH meter device.

The content of clay fraction was determined by Bouyoucos areometric method with Casagrande and Proszynski modification (Ryżak et al., 2009).

The content of organic carbon was measured with the use of Shimadzu’s SSM-5000A carbon analyzer.

The pseudo-total content of cadmium, lead, and zinc in the soils was measured. This is typical approach in case of monitoring works, as pseudo-total contents give an assessment of the maximum potentially soluble or mobile contents of metals (moreover, metals that are contaminants are usually not bound in silicates) (Relić et al., 2011). The extraction procedure was conducted based on US EPA standard and on our previous works (EPA, 1996; Miśkowiec & Olech, 2020; Miśkowiec et al., 2016). Approximately 1.5 g of each sample (dried previously to the constant mass in 105 °C), weighed to the nearest 0.0001 g, was digested with the mixture of the concentrated (65%) nitric acid and 8.8-M solution of hydrogen peroxide (solid to solution ratio 1:10) in a microwave oven. The high-performance digestion unit (MAGNUM II from Ertec, Wroclaw, Poland), with a compartment for a single PTFE digestion vessel designed for pressures up to 50 bar, served as the closed-pressurized microwave system.

The modified Bureau Communitaire de Reference (BCR) method (Rauret et al., 2000), successfully applied in our previous studies (Miśkowiec & Olech, 2020), was conducted for the so-called functional speciation analysis of the tested metals in soil. Four forms (FM) of metal binding in soil have been extracted. The first was the exchangeable one, easily soluble in an acid environment (FM1) and the most bioavailable; the second one was the form bound with iron and manganese hydroxides and oxides, prone to reducing (FM2); the third one was the form bound with organic matter, prone to oxidizing (FM3); and the fourth form, the residual part of metal, entrapped within the crystal structure of minerals (FM4). For the extraction of particular metal forms, the following chemicals were used: to extract the FM1 form, the 0.11 M acetic acid was applied, and for the second form (FM2) extraction, the 0.5 M hydroxylamine hydrochloride solution was used, whereas to extract the third form (FM3), the mixture of 8.8 M hydrogen peroxide and 1 M ammonium acetate of pH = 2 was applied. In the procedure of the FM4 form extraction, the mixture of concentrated HNO_3_ and 8.8 M hydrogen peroxide was applied in the high-performance digestion unit. For each sample, two independent replicates were performed, in the same way the blanks were measured for each set of analyses.

The solutions obtained from the digestion and from all the steps of BCR extraction were analyzed to determine the contents of particular metal with the flame atomic absorption spectrometry (F-AAS), using the Perkin Elmer apparatus AAnalyst 300. Based on the results of three independent measurements performed for each sample, the average values of cadmium, lead, and zinc concentration as well as the relative standard deviations were calculated. In order to validate the method for accuracy and precision, certified reference material (CRM044-50G TRACE METALS—SILT LOAM 1) was analyzed in analogous manner for corresponding elements. The recoveries were as follows: zinc—94%, lead—96%, cadmium—98%. The detection limit was determined as threefold standard deviation of repeated measurements of a blank solution and equaled for zinc 5 µg/dm^3^, lead 7 µg/dm^3^, and cadmium 1.5 µg/dm^3^. All calibration lines were linear, with a correlation coefficient higher than 0.994. The AAS sequence included a QC sample and a blank after 10 soil samples.

### Geoaccumulation index and potential ecological risk index calculations

The assessment of soil contamination with heavy metals may be carried out on the basis of appropriate indices. Two of the most popular are geoaccumulation index and potential ecological risk index (Kowalik et al., 2021). The geoaccumulation index (I_geo_) reflects both the natural variation in the heavy metal distribution and the impact of human activities (Wieczorek et al., 2018). The potential ecological risk index of particular toxicants (E_r_^i^) and potential ecological risk by the overall studied substances (PERI) consider additionally the toxicity of chemicals, to evaluate their potential impact on the environment (González-Valoys et al., 2021; Hakanson, 1980; Hu et al., 2021).

The geoaccumulation index (I_geo_) is a quantitative index used to study the degree of heavy metal pollution in soils and sediments (Amaro-Espejo et al., 2020; Fazekašová et al., 2021; Gąsiorek et al., 2017). It is calculated as follows:

$$\mathop I\nolimits_{geo} = \log_{2} \frac{{C_{n} }}{{1.5B_{n} }},$$

where *C*_*n*_ (mg*kg^−1^) is the average content of heavy metal in the soil and B_n_ (mg*kg^−1^) is the geochemical background value of heavy metal in the region (Kabata-Pendias, 2010). The 1.5 coefficient takes into account lithological variability of soils. The geoaccumulation index has seven degrees, from 0 to 6, with the sixth degree reflecting almost 100-fold enrichment of the soil with the examined element in relation to the background value (Förstner et al., 1990).

Potential ecological risk index (PERI) is one of the most commonly used, referred to Hakanson (Hakanson, 1980) method with the following formula:

$${E}_{r}^{i}={T}_{r}^{i}*\frac{{C}_{s}^{i}}{{C}_{n}^{i}},$$

$$PERI= {\sum }_{i=1}^{n}{E}_{r}^{i},$$

where *PERI* is a comprehensive potential ecological risk index, *E*^*i*^_*r*_ is the potential ecological risk associated with the particular heavy metal in the soils studied, *C*^*i*^_*s*_ (mg*kg^−1^) is the determined concentration of single metal in the soil, and *C*^*i*^_*n*_ (mg*kg^−1^) is the background value characteristic for particular area. *T*^*i*^_*r*_ is defined as the toxic response factor. The values of *T*^*i*^_*r*_ vary depending on type of metal and in case of studied metals equal: 30 for cadmium, 5 for lead, and 1 for zinc. Studies conducted by different authors have defined five classes of Er_i_ and four classes of PERI depending on risk level (Baran et al., 2017; Hakanson, 1980; Wieczorek et al., 2018). The background values for both indices were as follows: Zn – 40 mg*kg^−1^, Pb – 18 mg*kg^−1^, Cd – 0.22 mg*kg^−1^. These values are described as averages for unpolluted soils in Poland, e.g., by Kabata-Pendias and Pendias and by Lis and Pasieczna (Kabata-Pendias & Pendias, 1999; Lis & Pasieczna, 1995).

The map depicted in the Fig. 1 is based on data from ^©^2022 Google and prepared using Google tools and Corel PhotoPaint X8. The source of the map can be found in Google Maps (2022). OriginPro 2016 software was used to prepare 3D chart (Fig. 3) as well as for the statistical calculations (OriginLab, Northampton, MA).

## Results and discussion

The studied soil physicochemical parameters in the presented area do not differ drastically between samples, regardless on the slope exposition. The average pH value (4.2 ± 0.6) is typical for carbonate-free soils (Kicińska, 2016). The mean content of clay fraction was about 7% and organic carbon content ranged between 1.5 and 4%. This information allows one to classify all the soils as being “light.” Soils of this type (acidic, poor in both organic matter and clay fraction) are in general susceptible for the external factors disrupting homeostasis including chemical contamination (Miśkowiec et al., 2015).

The total content of cadmium lead and zinc in the soil samples, depending on the slope direction as well as the basic statistical parameters, is presented in Table 1. The observed mean levels of cadmium, lead, and zinc content in the topsoil of the windward side were distinctly higher than the assumed average concentrations in uncontaminated soils (which equal 0.41, 27, and 70 mg*kg^−1^ respectively), whereas on leeward side, the appropriate mean values except for lead content were lower than the average concentrations quoted above (Kabata-Pendias, 2010). The results indicating an increased content of heavy metals in the analyzed soils are consistent with scientific reports in which increased concentrations of the metallic elements (especially cadmium) in mosses and lichens of the studied region were noted (Godzik, 1991; Klimek et al., 2015).

**Table 1:** The basic statistical parameters of the measured concentrations of Cd, Pb, and Zn in the soil samples, depending on the slope direction

	Cd (mg*kg^**−1**^** of dry mass)**		Pb (mg*kg^**−1**^** of dry mass)**		Zn (mg*kg^**−1**^** of dry mass)**	
	**Windward**	**Leeward**	**Windward**	**Leeward**	**Windward**	**Leeward**
Mean	0.79	0.22	57.5	31.9	99.9	56.4
Median	0.75	0.16	54.5	26.2	95.8	53.8
Minimum	0.22	0.04	16.3	13.8	46.4	35.2
Maximum	1.55	0.56	112.7	63.8	189.1	80.4
SD	0.36	0.17	21.5	15.6	30.7	12.4
Skewness	0.86	0.55	0.27	0.89	1.09	0.12
Kurtosis	0.42	−1.16	0.50	−0.44	1.96	−1.02

The characteristic trait of all the heavy metals was positive and high values of skewness, which are rather typical and indicate a small share of clearly high metal concentrations. However, the platokurtic character of distribution of metal concentration on the leeward side and leptokurtic on the windward side may be another evidence of the measurable differences in the pollutants inflow (Wieczorek et al., 2018).

The calculated indices of geoaccumulation and potential ecological risk are presented in Table 2. The mean values of the pollution indices show moderate to low pollution of the studied areas with the particular heavy metals. However, as it can be noticed, the differences between windward and leeward sides are even more pronounced than for the absolute values of metals’ concentration. While the leeward sides of the slopes in most cases may be classified as uncontaminated with a low ecological risk, the indicators are significantly increased on the windward side of the studied mountain massif. This trend is particularly visible in the case of cadmium. At several windward measuring points, the I_geo_ values for Pb reached and for Cd even exceed 2, which means significant contamination. In the case of cadmium, it is particularly important from an ecotoxicological point of view, as it represents a high potential ecological risk. In over 22% of cases, the potential environmental risk is described at least as considerable (Er_i_ > 80) and at 12% of cases as high (Er_i_ > 160)—all on the windward side of the massif. Thus, cadmium’s contribution is the most significant in the PERI calculation—in 24% of cases, the potential ecological risk with the all three metals is depicted as at least considerable (PERI > 130).

**Table 2:** The geoaccumulation indices and potential risk indices of Cd, Pb, and Zn depending on slope wind exposition

	**Risk factor**	**Windward side**		**Leeward side**	
		**Mean value, range**	**Description of the mean level of contamination/risk**	**Mean value, range**	**Description of the mean level of contamination/risk**
Cd	I_geo_	1.1 (−0.6–2.4)	Moderately contaminated (1 < I_geo_ < 2)	−1.1 (−3.0–0.7)	Practically uncontaminated (I_geo_ < 0)
	Er_i_	108 (30–239)	Considerable risk (80 < Er_i_ < 160)	29.5 (5.5–76)	Low risk (Er_i_ < 40)
Pb	I_geo_	1.1 (−0.7–2.0)	Moderately contaminated (1 < I_geo_ < 2)	0.1 (−1.0–1.2)	Uncontaminated to moderately contaminated (0 < I_geo_ < 1)
	Er_i_	16 (4.5–31)	Low risk (Er_i_ < 40)	8.9 (3.8–18)	Low risk (Er_i_ < 40)
Zn	I_geo_	0.4 (−0.6–1.4)	Uncontaminated to moderately contaminated (0 < I_geo_ < 1)	−0.4 (−1.0–0.2)	Practically uncontaminated (I_geo_ < 0)
	Er_i_	2.1 (1.0–3.9)	Low risk (Er_i_ < 40)	1.2 (0.7–1.7)	Low risk (Er_i_ < 40)
PERI		126 (48–274)	Moderate risk (65 < PERI < 130)	39.5 (13–84)	Low risk (PERI < 65)

To exclude the hypothesis about the impact of parent rock on metal concentration in the topsoil and indirectly on calculated indices, the content of studied metals in substratum/bedrock horizon of five sampling points was measured. The obtained values have been typical for the carbonate free rock of flysh, and equaled as follows: Zn 22.6–45.9 mg*kg^−1^, Pb 5.8–11 mg*kg^−1^, Cd below detection limit to 0.3 mg*kg^−1^. The detailed results are presented in the supplementary materials (Table S2). Such values of metal content in bedrock are similar to those obtained by other authors in the neighboring areas and are considered as low (Józefowska et al., 2014; Panek, 1991). Thus, the presented results show that the bedrock is not responsible for the enrichment of the topsoil in this case.

**Fig. 2:**
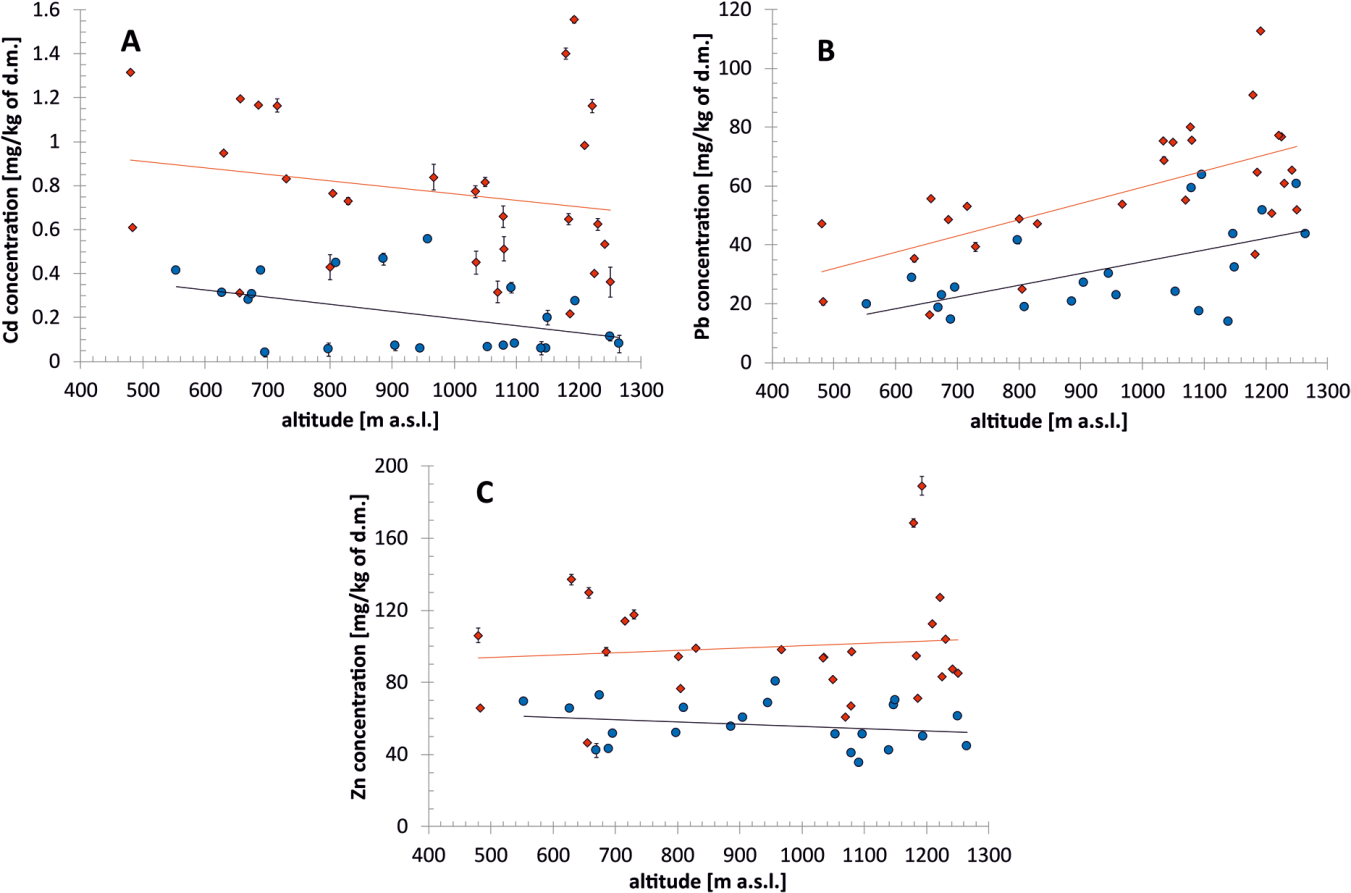
The dependences of metal concentration in Gorce soils on the altitude and wind exposition of the slope (red squares – windward, blue circles – leeward). **A** Cd, **B** Pb, **C** Zn. The equations of regression are as follows: cadmium—windward side: *y* =  −0.0003x + 1.0592, leeward side: *y* =  −0.0003x + 0.522; lead—windward side: *y* = 0.0551x + 4.524, leeward side: *y* = 0.0399x −5.6277; zinc—windward side: *y* = 0.0129x + 87.529, leeward side: *y* =  −0.0123x + 67.977

In Fig. 2, the dependence of the pseudo-total metal concentration on the altitude and wind exposition of the slope is presented. By analyzing linear regressions, a slight upward trend, coherent with the predictions, can be observed for zinc on the windward side and lead on windward and leeward side, with the determination coefficients 0.0129, 0.0551, and 0.0399, respectively (Fig. 2B, C). The slight downward trend in the case of cadmium (− 0.0003 in both sides—Fig. 2A) is most likely due to the large dispersion of the results above 1000 m above sea level. This dispersion may be due to the fact that the concentrations of this metal in samples are generally the lowest of the three metals tested and may be subject to the largest random fluctuations. Therefore, the cadmium content in the soil may be influenced to a greater extent by local factors—practically unmeasurable and unpredictable, such as varied plant cover, slightly different use of the studied meadows and glades (sheep grazing, cattle grazing, meadows mowing), or different degrees of slope inclination and the influence of surrounding forests. However, it should be underlined that the values of all determination coefficients of the regression lines close to zero indicate only a slight gradation of pollutant concentrations with height and do not allow for unequivocal determination of the dependence of the observed heavy metal content on the height above sea level. Gorce is most likely a mountain group too low for such trends to be clearly visible. However, the differences in metal concentration depending on the slope exposure are visible in all three cases regardless of the altitude and the dispersion of the results.

The significance of the above-described differences has been confirmed by the statistical Welch’s *t*-test. The parameters of the test are presented in Table 3. According to the calculations, the zero hypothesis about lack of differences between windward and leeward side in cases of all three metals can be discarded with the probability 0.99.

**Table 3:** The parameters of the Welch’s *t*-test of the similarity of two populations within analysis of the particular metals

	**Windward side**	**Leeward side**	**Windward side**	**Leeward side**	**Windward side**	**Leeward side**
The element	Cd		Pb		Zn	
Mean conc. value (mg/kg)	0.79	0.22	57.5	31.9	99.9	56.4
Samples number	28	22	28	22	28	22
Variance	0.13	0.03	462	244	945	1
**t calc. statistics**	**6.88**		**4.87**		**6.83**	
Calculated approx. degrees of freedom	37		47		37	
**t tab. value from the *t*-distribution table for the calculated number of degrees of freedom (*p* value = 0.01)**	**2.715**		**2.685**		**2.715**	
*t* comparison	t_calc_ > t_tabl_		t_calc_ > t_tabl_		t_calc_ > t_tabl_	
H0 hypothesis	Rejected		Rejected		Rejected	

**Fig. 3:**
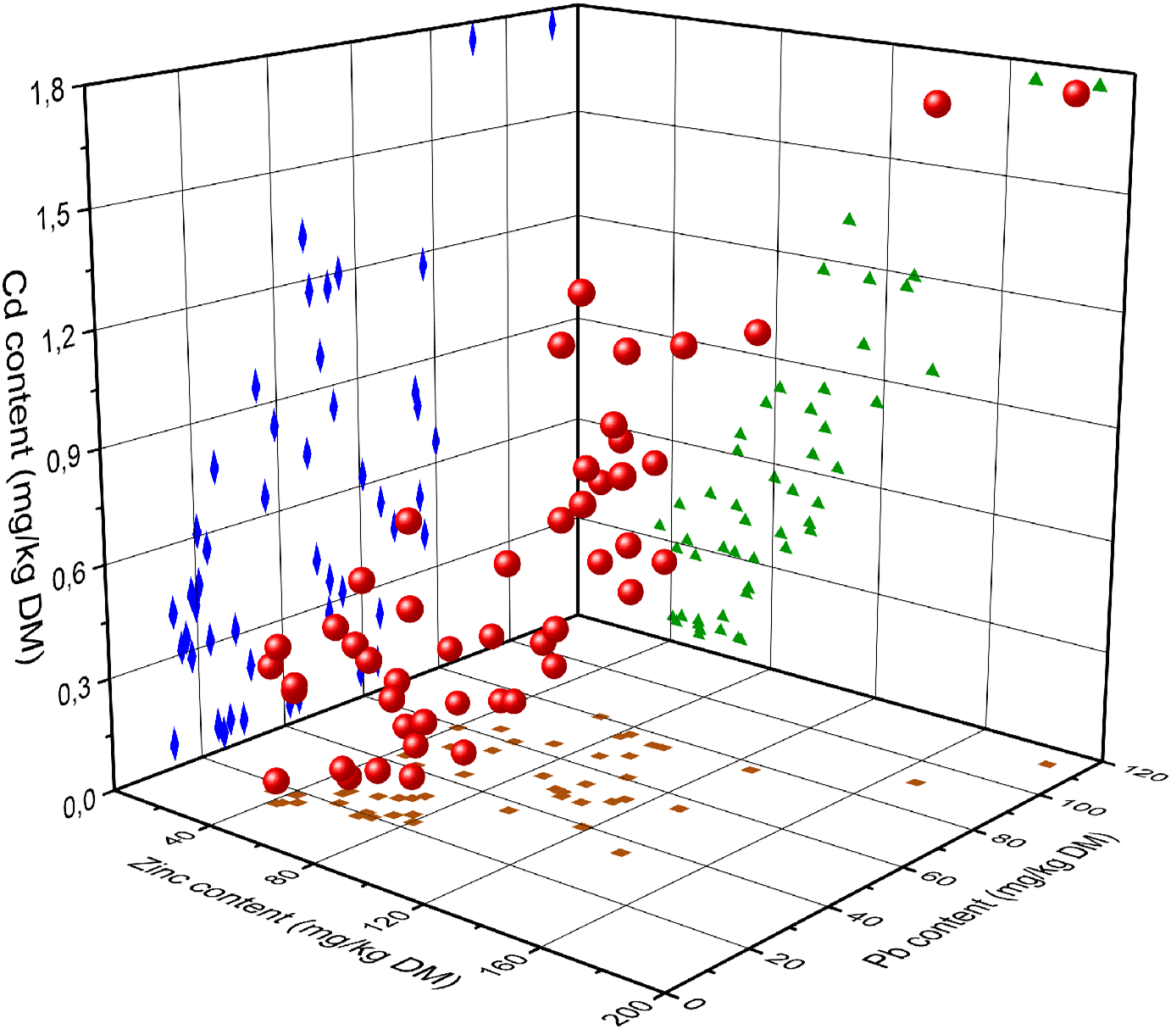
3D chart depicting the dependences between contents of Zn, Pb, and Cd in the soils studied (red balls). Diamonds, triangles, and squares are the projections of the chart on individual 2D planes, showing the relationships between particular two parameters: Cd/Pb (diamonds), Zn/Pb (squares), and Cd/Zn (triangles). The calculated Spearman correlation factors are as follows: Cd/Pb 0.45, Pb/Zn 0.52, Cd/Zn 0.82

In addition, the correlations between concentration of all three metals have been calculated and are depicted in the Fig. 3. Due to the skewness of data and deviation from the normal distribution (confirmed with Shapiro–Wilk test), Spearman correlation factor has been calculated. The appropriate values of correlation factors are as follows: Cd/Pb 0.45, Pb/Zn 0.52, Cd/Zn 0.82. The presented correlation values confirm the assumption of the common source of contamination in this case. However, the factor values are lower than one may have expected and were observed in different components of the environment, located closer to the potential emitters (Miśkowiec, 2018b; Miśkowiec et al., 2015). Thus, the local variations in concentrations must be taken into account, as they appear to have an increasing effect on the concentration of metals, which is probably already due to a relatively large distance from emission sources. By analyzing Figs. 2 and 3 and Table 3 together, as well as data on metal content in the parent rock, it can be seen that the heavy metals in topsoil have a common origin and this is most likely the influence of the Jaworznicko-Chrzanowski Industrial Belt and the Silesian conurbation as well as Kraków—Nowa Huta steelwork to some extent. However, these contents are low enough to observe local fluctuations in the concentrations of the tested metals. In the case of Gorce, the height above sea level as a function of the concentration of toxicants evidently plays a secondary role in comparison to the orientation of the slope.

**Fig. 4:**
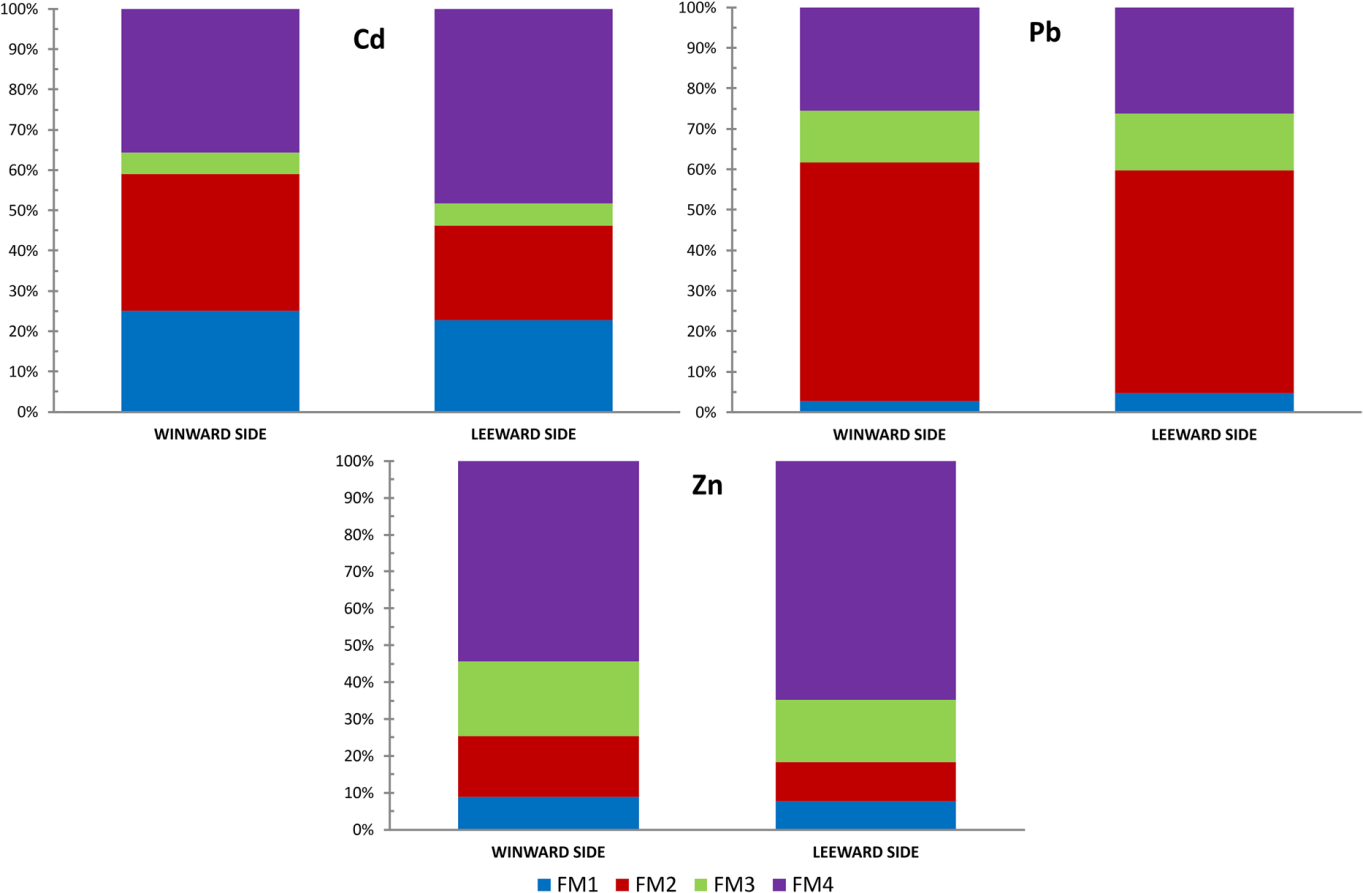
The average relative content of zinc, lead, and cadmium in the soils studied, with the divisions on windward and leeward side and in four metal forms gained with the BCR extraction method. FM1, water and light acids soluble part; FM2, the form bound to iron and manganese oxides/hydroxides; FM3, the form bound to organic matter; FM4, the residual part of metal, entrapped within the crystal structure of minerals

The BCR sequential extraction gave the picture of the distribution of particular forms of metals in the soils studied. The appropriate values were averaged for windward and leeward side and are depicted in the Fig. 4. As it can be seen, the distribution of particular forms differs to a small extent depending on the hill sides. The share of the residual fraction of cadmium in the leeward was higher than in the windward side, amounting to 48% and 35%, respectively. However, the high proportion of forms easily absorbed by plants (over 20% of the total amount of metal in both sides) is also noticeable. In case of lead, the changes are minimal in favor of the residual and exchangeable forms on the leeward side. In case of zinc, similarly like in case of cadmium, the increase by approximately 15% of the share of the residual form in the leeward side is visible. The increase of share of the so-called residual forms of metals in the soils from the leeward side of the range with a simultaneous decrease in the absolute content clearly proves the hindered inflow of new pollutants into these areas, in contrast to the areas located on the windward side. This phenomenon, which is generally observable in higher mountains as the Alps (Zechmeister, 1995) or Andes (Romo-Kröger & Llona, 1993; Santos-Francés et al., 2017), was usually considered negligible in the lower mountains. The results show, however, that regardless of the absolute height of the mountain massif, it can be a considerable barrier to atmospheric pollution.

The results prove the existence of statistically significant differences in the concentrations of all three tested heavy metals between the windward and leeward sides of the studied mountain massif. The observed lack of a clear trend of concentration changes with height is interesting. On the windward side, the incoming pollutants are distributed relatively evenly, regardless of the absolute height, and there is likewise no vertical differentiation of toxicants on the leeward side. Thus, the studied mountain range is probably high enough to constitute a barrier to the spread of pollutants, but too low to observe such a vertical variability.

It should be noted, however, that the relatively high scatter of the results both on the windward and leeward sides, regardless of their skewness and kurtosis, may already be due to a certain “blurring” of the examined differences. This is most likely due to both: the already signaled long distance from the main sources of pollution (the mining and smelting plant in Bukowno, hard coal mines in Silesia, steelwork in Kraków-Nowa Huta) and the general decrease in the intensity of emissions of harmful substances into the atmosphere by industrial sources, observed over the last three decades (Chodor et al., 2018). It is also confirmed by relatively low values of the tested pollution indices (except for the values for cadmium on the windward side). On the other hand, in view of the decline in industrial emissions, local sources of air pollution are gaining in importance in the overall balance. In the case of the discussed area, an important role is played mainly by the so-called low emission, i.e., burning poor-quality coal in households. It is visible, for example, in the Nowy Targ basin—the plain located south of the Gorce Mountains, with the dominant city of Nowy Targ. In winter, this region often experiences temperature inversion, which, combined with low emissions, causes the formation of London-type smog (Czarnecka et al., 2021; Palarz et al., 2017). The formed suspensions may, in the long run, to some extent affect the content of the tested heavy metals also in the soils of Gorce Mountains.

## Conclusions

In this study, one postulates the existence of measurable pollution with cadmium, lead, and zinc of the mountain soils placed over 100 km from the potential sources of contamination. Regardless of the low both prominence (up to 1310 m a.s.l.) and average relative high over the surrounded areas (approximately 700 m), the studied mountain massif turned out to be barrier of measurable impact on the spreading of atmospheric pollutants. While the concentrations of studied heavy metals in the area are not alarmingly high, it differs statistically significantly between the windward and leeward side of the massif. Thus, it turned out that even unremarkable mountain barrier may play a significant role in the spread of pollution. Moreover, the windward sides of mountains are in such a case much more vulnerable to contamination than would otherwise arise from simple contamination spread modeling, not taking into account the altitude and land relief. This fact may be particularly dangerous considering the low resistance of mountain soils to pollution and should be taken into account both in protection plans and in developing the increasingly popular traditional pastoral and agricultural economy caused by the development of tourism in the region.

## Supplementary Information

Below is the link to the electronic supplementary material.

Supplementary file1 (DOCX 2508 KB)

## Data Availability

This is the original data presented in this manuscript. If raw data is required, it will be provided on reasonable request.
