# Supplementary material for: The impact of the mountain barrier on the spread of heavy metal pollution on the example of Gorce Mountains, Southern Poland
Source: Environ Monit Assess. 2022 Aug 10;194(9):663. doi: 10.1007/s10661-022-10316-0 (PMC9365746; doi:10.1007/s10661-022-10316-0)
Supplement: Supplementary file 1 — Supplementary file1 (DOCX 2508 KB) [file 10661_2022_10316_MOESM1_ESM.docx]

Supplementary materials

*Table S1. Sampling points description, altitude, latitude, longitude and exposition*

|  | **#** | **Sampling point description** | **altitude [m a.s.l]** | **latitude** | **longitude** | **exposition of the sampling area** |
| --- | --- | --- | --- | --- | --- | --- |
| windward side | 1 | Over the Niedźwiedź village | 480 | 49°37'20''N | 20°05'11''E | N |
|  | 2 | Southern outskirt of the Mszana Dolna town | 483 | 49°39'45''N | 20°04'07''E | N |
|  | 3 | Over the Lubomierz village | 630 | 49°36'17''N | 20°12'32''E | N |
|  | 4 | Western slopes of the Piątkowa mountain | 655 | 49°34'47"N | 19°56'01"E | NW |
|  | 5 | Hucisko glade - forester's lodge area | 657 | 49°34'45''N | 20°05'09"E | N |
|  | 6 | Over the Konina village | 685 | 49°35'01''N | 20°08'02''E | N/NW |
|  | 7 | Trusiowka meadow - Kamienica creek valley | 716 | 49°35'03''N | 20°13'18''E | N |
|  | 8 | Wierch Młynne pass | 730 | 49°34'14''N | 20°17'39''E | N |
|  | 9 | Gąsiorowska glade lower part | 801 | 49°35'09''N | 20°05'33''E | N |
|  | 10 | Gąsiorowska glade upper part | 830 | 49°35'09"N | 20°05'33"E | N |
|  | 11 | Western slopes of the Kułakowy Wierch | 805 | 49°32'36''N | 19°58'08''E | W/NW |
|  | 12 | Suhora glade | 967 | 49°33'49''N | 20°04'08''E | N |
|  | 13 | Turbaczyk glade - lower part | 1035 | 49°34'57''N | 20°06'49''E | NW |
|  | 14 | Turbarczyk glade - upper part | 1050 | 49°34'48"N | 20°06'56"E | NW |
|  | 15 | Turbaczyk mountain | 1078 | 49°34'46''N | 20°06'57''E | NW |
|  | 16 | Spalone glade lower part | 1034 | 49°34'32''N | 20°06'53''E | N |
|  | 17 | Spalone glade upper part | 1070 | 49°34'27"N | 20°06'52"E | N |
|  | 18 | Obidowiec glade | 1080 | 49°33'33''N | 20°04'37''E | N |
|  | 19 | Gorc halls - upper part | 1183 | 49°33'42"N | 20°14'51"E | N/NE |
|  | 20 | Czoło Turbacza glade | 1179 | 49°33'18''N | 20°06'44''E | N |
|  | 21 | Northern slope of Czoło Turbacza mountain | 1192 | 49°33'17"N | 20°06'46"E | N |
|  | 22 | Mostownica glade - lower part | 1186 | 49°33'23''N | 20°08'09''E | N/NE |
|  | 23 | Mostownica glade - upper part | 1242 | 49°33'16''N | 20°07'51''E | N |
|  | 24 | Turbacz Hall - southern - east part | 1209 | 49°32'58''N | 20°07'01''E | N/NE |
|  | 25 | Wzorowa Hall | 1221 | 49°32'49''N | 20°07'41''E | N |
|  | 26 | Turbacz Hall, northern east part | 1225 | 49°33'06''N | 20°07'05''E | NW |
|  | 27 | Turbacz hall - central part | 1230 | 49°33'01"N | 20°06'51"E | N |
|  | 28 | Northern slope of the Turbacz mountain | 1250 | 49°32'47''N | 20°06'40''E | N |
| leeward side | 29 | Over the Łopuszna village | 554 | 49°28'43''N | 20°08'30''E | S |
|  | 30 | Meadows north of the Nowy Targ city | 627 | 49°29'35''N | 20°01'47''E | S |
|  | 31 | Over the Ostrowsko villige | 670 | 49°29'03''N | 20°05'58''E | S |
|  | 32 | Over the Knurów villige | 675 | 49°29'14"N | 20°10'33''E | S/SE |
|  | 33 | Meadows in the Ochotnica Górna village | 690 | 49°30'27''N | 20°13'01''E | S |
|  | 34 | over the hamlet of Łopuszna village - Zarębek Średni | 697 | 49°30'06''N | 20°08'25''E | S |
|  | 35 | Chowańcowa glade | 798 | 49°30'27''N | 20°08'24''E | SE |
|  | 36 | Knurowska pass | 810 | 49°30'21''N | 20°11'26''E | SE |
|  | 37 | Cyrlica glade upper part | 905 | 49°30'53''N | 20°06'57''E | SE |
|  | 38 | Cyrlica glade lower part | 886 | 49°30'50"N | 20°07'04"E | SE |
|  | 39 | Srokówki glade | 946 | 49°30'53''N | 20°08'28''E | SE |
|  | 40 | Kramaszka glade lower part | 958 | 49°30'55"N | 20°08'31"E | SE |
|  | 41 | Gorcowskie Halls 1 | 1150 | 49°33'30''N | 20°14'55''E | S |
|  | 42 | Gorcowskie Halls 2 | 1140 | 49°33'22''N | 20°14'58''E | S |
|  | 43 | Wysznia glade | 1054 | 49°31'10''N | 20°08'16''E | S |
|  | 44 | Jankówki lower part | 1080 | 49°31'26''N | 20°08'21''E | SE |
|  | 45 | Jankówki upper part | 1092 | 49°31'35"N | 20°08'27"E | SE |
|  | 46 | Giecka glade | 1097 | 49°33'23''N | 20°04'51''E | SE |
|  | 47 | Zielenica glade | 1148 | 49°31'41''N | 20°08'48''E | SE |
|  | 48 | Świderowa glade | 1195 | 49°32'12''N | 20°06'33''E | SE |
|  | 49 | Długa Hall southern part | 1250 | 49°32'42''N | 20°07'41''E | S/SE |
|  | 50 | Młyńska Hall | 1265 | 49°32'23''N | 20°09'01''E | S |

Table S2 – concentration of studied heavy metals in the substratum horizon of five representative sample collection points.

| # sampling point | Depth of sampling collection [cm] | Heavy metal content [mg*kg^-1^] | | |
| --- | --- | --- | --- | --- |
|  |  | Zn | Pb | Cd |
| 1 | Below 85 | 37.5 ±0.3 | 11±1 | 0.30±0.04 |
| 4 | Below 80 | 45.9 ±0.2 | 9.2±0.7 | 0.20±0.03 |
| 8 | Below 65 | 28.5 ±0.6 | 5.8±2.2 | Below detection limit |
| 31 | Below 80 | 39.2 ±0.3 | 8.8±1.5 | 0.25±0.04 |
| 36 | Below 85 | 22.6 ±0.4 | 10.0±0.6 | Below detection limit |


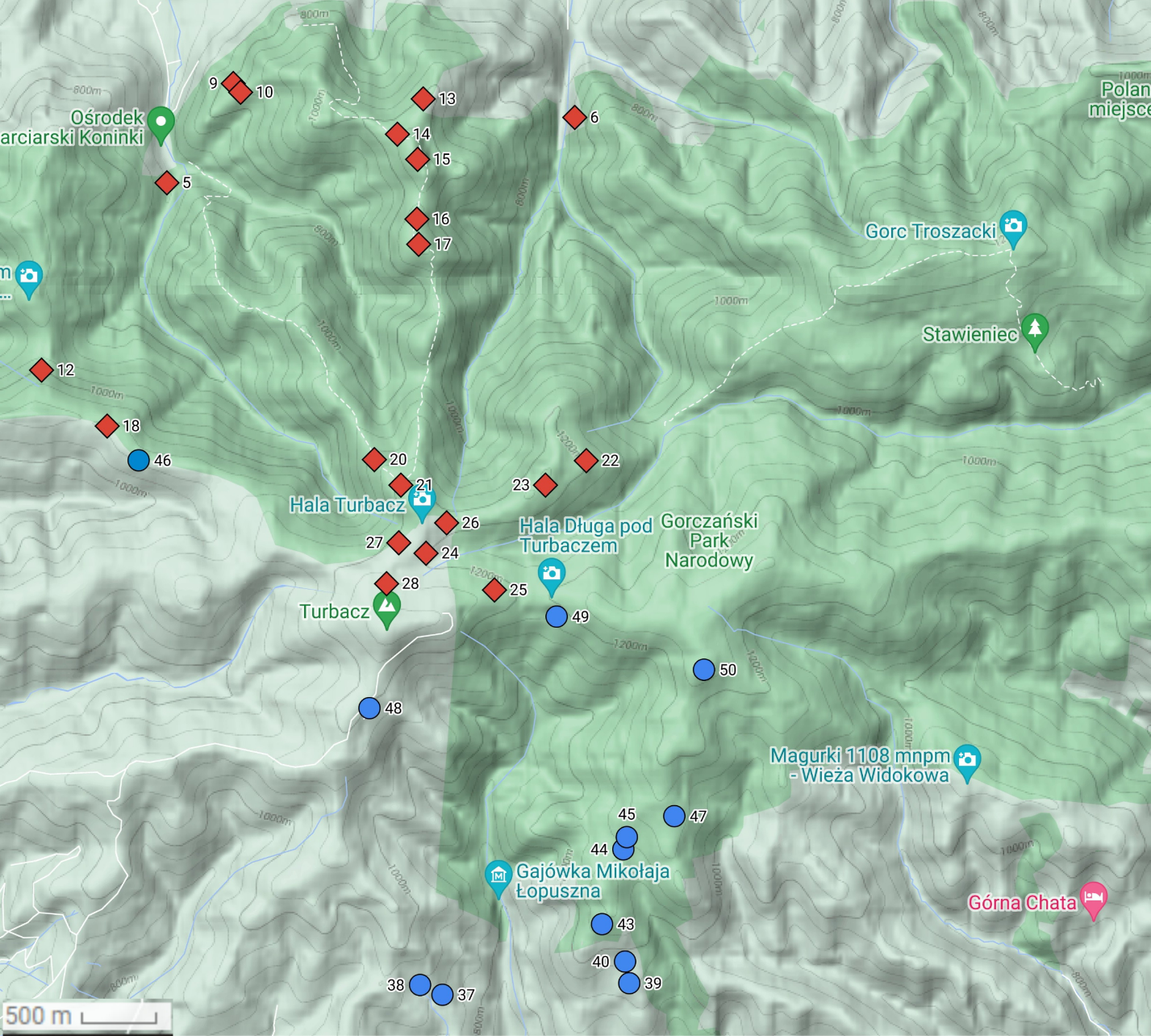


*Fig S1. The detailed map of the sampling points of the area with compacted measuring points.*
